# Thin-film PMUTs: a review of over 40 years of research

**DOI:** 10.1038/s41378-023-00555-7

**Published:** 2023-07-21

**Authors:** Kaustav Roy, Joshua En-Yuan Lee, Chengkuo Lee

**Affiliations:** 1 Department of Electrical and Computer Engineering, National University of Singapore, Singapore, 117583 Singapore; 2 Center for Intelligent Sensor and MEMS (CISM), National University of Singapore, Singapore, 117608 Singapore; 3 Institute of Microelectronics, A*STAR, Singapore, 117685 Singapore

**Keywords:** Electrical and electronic engineering, Electronic devices

## Abstract

Thin-film PMUTs have been important research topics among microultrasound experts, and a concise review on their research progress is reported herein. Through rigorous surveying, scrutinization, and perception, it has been determined that the work in this field began nearly 44 years ago with the primitive development of functional piezoelectric thin-film materials. To date, there are three major companies commercializing thin-film PMUTs on a bulk scale. This commercialization illustrates the extensive contributions made by more than 70 different centers, research institutes, and agencies across 4 different continents regarding the vast development of these devices’ design, manufacturing, and function. This review covers these important contributions in a short yet comprehensive manner; in particular, this paper educates readers about the global PMUT outlook, their governing design principles, their manufacturing methods, nonconventional yet useful PMUT designs, and category-wise applications. Crucial comparison charts of thin-film piezoelectric material used in PMUTs, and their categorically targeted applications are depicted and discussed to enlighten any MEMS designer who plans to work with PMUTs. Moreover, each relevant section features clear future predictions based on the author’s past knowledge and expertise in this field of research and on the findings of a careful literature survey. In short, this review is a one-stop time-efficient guide for anyone interested in learning about these small devices.

## Introduction

The field of transducers has undergone several revolutions. One of those reforms was the creation of nanotechnology, which enabled the making of very small transducers with submicron dimensions. This development and extensive material research gave birth to microelectromechanical systems (MEMS). Soon, the macro world of sensors shifted to the microscale, leading to an ever-increasing demand in the field. Today, most smartphones have MEMS inertial systems inside. MEMS has also revolutionized the field of ultrasound, leading to the creation of very small ultrasound transducers. These transducers are popularly known as micromachined ultrasound transducers (MUTs), and they can be broadly classified as capacitive MUTs (CMUTs)^1^ and piezoelectric MUTs (PMUTs).

The key component of a MUT is a suspended micro-diaphragm. A CMUT’s diaphragm consists of either a single layer of a conducting material or a single layer of a nonconducting material, both with an electrode layer, that is suspended generally with a 0.5 µm to 2 µm gap from the grounded substrate, which is electrostatically actuated with an alternating current (AC) electric field. Alternatively, a PMUT’s diaphragm consists of at least four layers, including a passive layer and a piezoelectric layer sandwiched between metal electrodes that can be dynamically actuated with AC.

Although there are existing reviews on PMUTs^2,3^ that provide an understanding of the subject, most of the important information about the topic has not been reported. Some examples of this important information are (a) the historical evolution of PMUTs, notable PMUT hotspots, publication statistics, and PMUT commercialization, (b) PMUT’s working principle and design maps, (c) special PMUTs with enhanced capabilities, including structurally modified PMUTs and flexible/stretchable PMUTs and (d) specific applications of PMUTs as transmitters, receivers, and transceivers. Above all, an important definition of PMUTs in terms of the thickness of the piezoelectric layer is missing in the existing reviews; without this definition, it is difficult to classify PMUTs into thin-film or thick-film categories. This review has been prepared to address this missing information, and it serves to provide a holistic overview of the past, present, and future research in this field. It starts with the PMUT evolution characteristics, followed by the design basics, novel piezoelectric thin films along with methods to fabricate PMUTs. The paper subsequently continues with an explanation of special PMUTs with unconventional designs and their research implications, followed by a vivid description of the major applications using them for constructing a transmitter, receiver, or transceiver. Two critical comparison charts of various thin-film piezoelectric materials and PMUT applications have been tabulated, which can easily give the reader a sense of their quantifiable qualities.

## PMUT evolution: past to present

### About thin-film PMUTs

#### What are PMUTs?

**Fig. 1:**
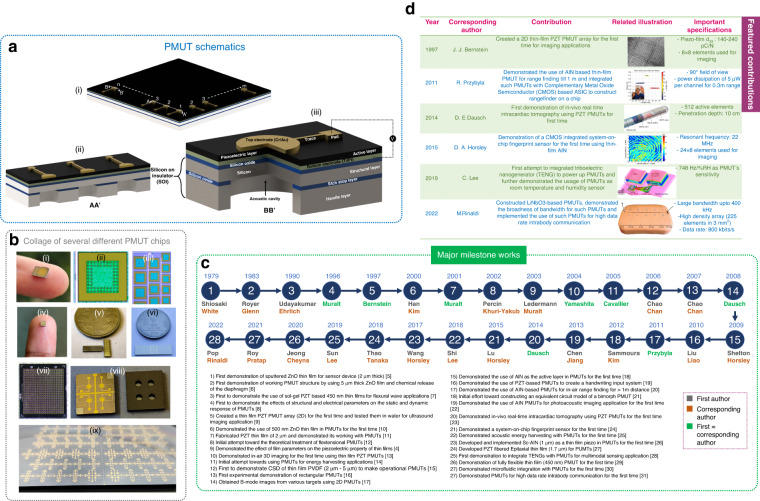
PMUTs and their evolution. **a** Schematic of a bulk micromachined PMUT: (i) typical 2D PMUT die with m x n elements; (ii) cross-section of a PMUT linear array; and (iii) single-cell PMUT’s 3D quarter cut section showing various important layers. **b** Collage of different fabricated PMUT chips; (i) 4.5 mm circular dual-electrode PMUT for communication applications; (ii) single element perforated PMUT for high-quality factor applications; (iii) multifrequency PMUT array with elements of different dimensions; (iv) single element, multidevice PMUT for functional spectroscopy applications; (v) 16-element 250 µm PMUT linear array; (vi) PMUT used for microfluidic integrated sensing applications; (vii) high-density PMUT 2D array for endoscopy; (viii) dual electrode 140 kHz PMUT for liquid density sensing application; and (ix) undiced array of multiple sized PMUTs. **c** Pictorial representation of the major milestone works in the area of PMUTs from 1979 to 2022. **d** Featured contributions with PMUTs selected from the milestone works. All pictures have been adapted with permission

PMUTs are micromembrane ultrasound transducers backed by an acoustic cavity that can be nanofabricated in various shapes and dimensions. A PMUT’s transduction mechanism is governed by thin-film piezoelectricity, causing necessary vibrations. PMUTs are generally operated at their resonant frequencies and can function as transmitters/actuators, receivers/sensors, and transceivers. As a transducer, PMUTs have three advantages over commercially available bulk thick-film ultrasound transducers: (a) they require relatively low actuation voltages to produce a unit acoustic pressure per unit area (Pa/V/mm^2^), making them suitable for portable low-power applications, (b) they can operate both in air and water due to an efficient impedance matching with the surroundings, and (c) they can be made in tiny form factors, thereby enhancing their suitability for space-constrained applications. Figure 1a(i) represents a PMUT die with elements arranged in 2D m x n arrays. Figure 1a(ii) shows a portion (AA’) of a linear PMUT array cut from the die. One of the elements in the die is cut (BB’) in the form of a three-quarter cross-section and is depicted in Fig. 1a(iii). The figure shows all the layer constituents of a typical PMUT fabricated by the bulk micromachining of silicon. Figure 1b shows a collage of different PMUT chips fabricated by the author’s groups, all with unique applicability.

#### Piezoelectric active layer thickness in thin-film PMUTs

PMUTs function according to the lateral dynamic strain developed in the piezoelectric thin film, and they perform best if the thickness of the film is below a critical limit; above this limit, the transverse strain cross-interference becomes noticeable. There are works that experimentally define this limit as 3 µm in a lead zirconate titanate (PZT) {100}-textured thin film^4^, above which there is a significant decay in the lateral thin-film-based (*31*,f) piezoelectric coefficient. Thus, in this review, only relevant articles that satisfy this criterion have been included.

### Historical development

The first spark toward PMUT creation dates back to 1979, when Shiosaki et al.^5^ developed thin-film sputtered zinc oxide (ZnO) for sensors. Four years later, Royer et al.^6^ demonstrated a working PMUT structure by using thin-film ZnO through the chemical release of the PMUT diaphragm. In 1990, Udayakumar et al.^7^ used sol-gel (PZT) thin films for flexural wave applications, followed by Muralt et al., who demonstrated the effects of the membrane structure and electrical parameters of thin-film PZT on PMUT response in 1996^8^. Bernstein^9^ in 1997 created a thin-film PZT PMUT array for in-water ultrasound imaging. In 2000, Han et al.^10^ fabricated a ZnO film with a 500 nm thickness in a PMUT for the first time, which was the thinnest film till then. In 2001, Muralt^11^ fabricated a 2 µm thin film for a PMUT; then, Percin et al.^12^ attempted to theoretically treat flextensional PMUTs in 2002. In 2003, Ledermann et al.^4^ demonstrated the effects of film parameters on the piezoelectricity characteristics of thin films; Yamashita demonstrated in-air 3D imaging for the first time using thin-film PZT PMUTs^13^ in 2004. Cavallier et al.^14^ demonstrated energy harvesting applications using PMUTs in 2005. In 2006, Chao et al.^15^ demonstrated chemical solution deposition (CSD) of thin-film polyvinylidene fluoride (PVDF) to make operational PMUTs. This work was the first time a polymer piezoelectric material was used in PMUTs. Afterward, in 2007, Chao et al. reported rectangular PMUTs^16^, followed by Dausch, who obtained B-mode ultrasound images from various targets using a 2D PMUT array^17^ in 2008. In 2009, Shelton first proposed the use of aluminum nitride (AlN) thin films as the active layer in a PMUT^18^, leading to the creation of complementary metal–oxide semiconductor (CMOS)-compatible PMUT arrays. This work was followed by Liu et al. proposing a handwriting input system using PZT-based PMUTs^19^ in 2010. Subsequently, Przybyla^20^ reported on the use of AlN PMUTs for in-air range finding at >1 m distances in 2011. In 2012, there was an initial effort toward constructing an equivalent circuit model of a bimorph PMUT from Sammoura et al.^21^. In 2013, Chen et al. demonstrated the use of AlN PMUTs for photoacoustic imaging applications for the first time^22^. Dausch demonstrated in vivo real-time intracardiac tomography using PZT PMUTs in 2014^23^, which is considered the major in-body medical ultrasound imaging application demonstrated to date. In 2015, Lu et al.^24^ demonstrated a system-on-chip fingerprint sensor, which is one of the most important applications with relevance to consumer electronics. In 2016, Shi et al.^25^ comprehensively demonstrated acoustic energy harvesting with PMUTs, followed by Wang et al. developing scandium-doped AlN (Sc-AlN) for PMUTs for the first time in 2017^26^. Subsequently, in 2018, Thao et al. developed PZT-fibered epitaxial thin-film PMUTs^27^, which was followed by the development of energy-efficient triboelectric nanogenerator (TENG)-powered PMUTs for multimodal sensing applications by Sun et al.^28^ in 2019. In 2020, Jeong et al.^29^ developed a fabrication flow for flexible thin-film PMUTs, which has relevance in wearable medical applications. In 2021, Roy et al.^30^ developed a microfluidic integrated platform with PMUTs and demonstrated fluid density sensing, followed by applying PMUTs for high-data rate intrabody communication by Pop et al. for the first time in 2022^31^. Thus, by observing PMUT history, it is found that there has been massive progress in terms of material development, innovative PMUT designs and applications. The pictorial representation of the milestone works is depicted in Fig. 1c. Out of the milestone works, some further contributions have been tabulated as featured contributions in the attached 2D list (see Fig. 1d).

### PMUT hotspots and publication statistics

**Fig. 2:**
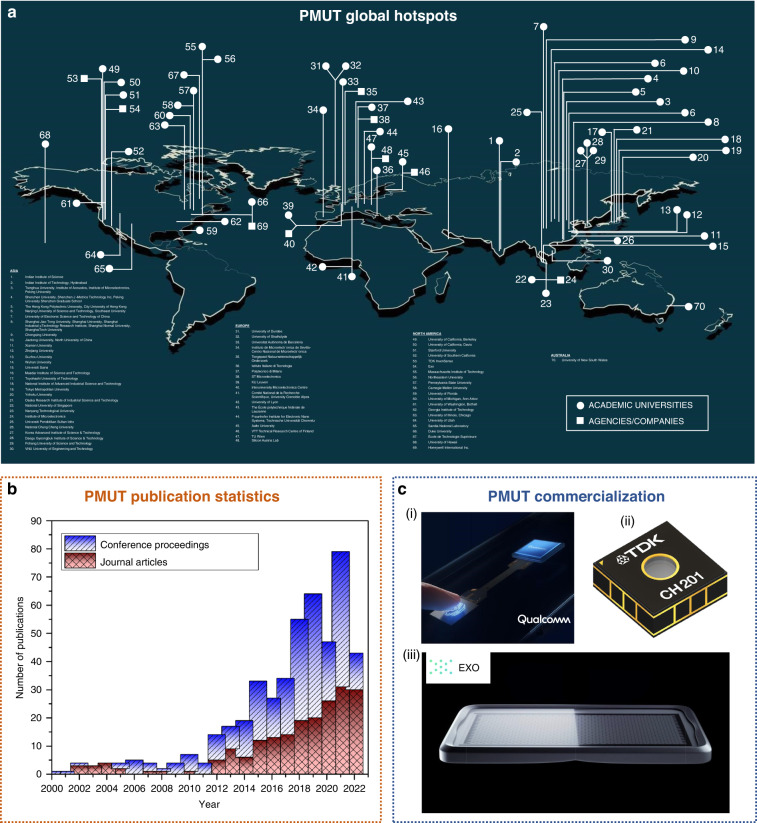
PMUT global development scenario. **a** PMUT research locations showing the active involvement of 4 continents: Asia, Europe, North America, and Australia. **b** PMUT publication statistics from 2001 to 2022. **c** Major companies commercializing PMUTs. (i) 3D sonic sensor used as an in-display fingerprint sensor developed by Qualcomm Inc. (ii) CH201, which is a 5 m PMUT rangefinder commercialized by TDK Inc. (iii) Cello, which is a handheld portable PMUT based imaging probe developed by Exo Inc. All pictures have been adapted with permission

The literature reveals nearly 70 places worldwide across 4 continents (in terms of academic universities and agencies/companies) working on PMUTs. Regarding the number of locations with extensive PMUT research, Asia has 30, Europe has 18, North America has 21 and Australia has 1 PMUT hotspot(s) respectively (Fig. 2a). Publication-wise, the number of research articles has increased exponentially, from 14 published articles in 2012 (Fig. 2b) to 79 in 2021, which indicates an ever-increasing demand for researchers to work in this domain to create novelties.

### PMUT commercialization

PMUTs have been commercialized by three companies (Fig. 2c): Qualcomm, TDK, and Exo. Qualcomm has developed the first commercial in-display PMUTs (called 3D sonic sensor) to map 3D fingerprints. TDK has commercialized application-specific integrated circuit (ASIC)-bonded PMUTs titled CH101 and CH201 that are suitable for range finding to 1.2 m and 5 m, respectively. Exo has developed a handheld prototype called Cello, containing 4096 low-powered PMUTs for multiharmonic imaging.

### Author’s sectional predictions

There has been rapid growth in the number of popular research locations, publication articles, and PMUT-based companies in the last five years. According to the growth trends, it is predicted that by 2025, there will be at least 30 new hotspots for PMUTs, with the total number reaching 100. As seen in Fig. 2, Asia will continue to be the leading continent researching PMUTs, with at least 10 new institutes becoming involved in PMUT research in the next 5 years from China. Articles will continue to increase monotonically, with more than 300 research articles and 10 review articles published in the next 5 years. The increase in the number of new companies is unpredictable and complicated, but it is believed that there will be at least 3 more major investments worldwide in this direction. Since all research features phases of growth, saturation, and decay, PMUTs will exhibit this trend; it is predicted that research in this direction will hit saturation in the next 7–10 years, thereby following decay.

## PMUT design basics

### PMUT structural classification

#### Structural representation of PMUTs

**Fig. 3:**
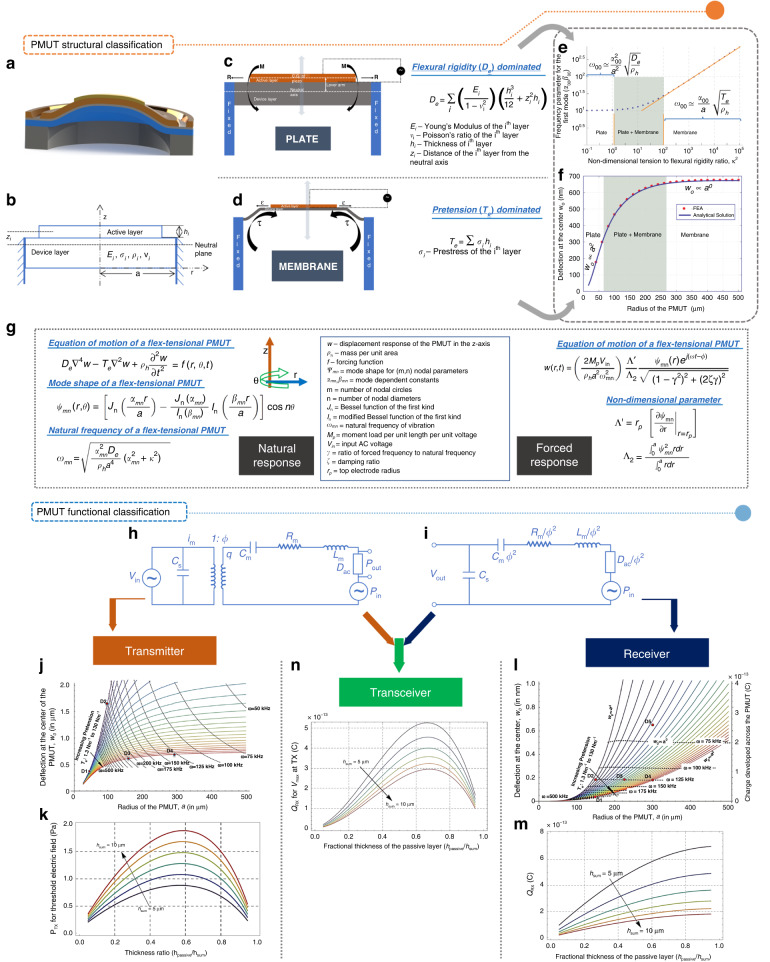
PMUT design basics which can be divided into two classes based on PMUT structure and function. **a** Snapshot of a vibrating PMUT idealized by a modified parabolic shape. **b** Important structural layers of a PMUT – the device layer and the active piezoelectric layer. **c** Schematic of the working mechanism of an equivalent flexural rigidity dominated plate type PMUT. **d** Schematic of the working mechanism of a tension dominated membrane type PMUT. **e** Dependence of frequency parameter on kappa squared clearly showing three different working regimes^32^. **f** Dependence of a PMUT’s central deflection on the radius which also depicts three different working regimes. **g** Basic equations governing the vibration of a PMUT. **h** Lumped parameter model of a PMUT transmitter. **i** Lumped parameter model of a PMUT receiver. **j** A PMUT as a transmitter in the size-tension subspace^32^. **k** A PMUT as a transmitter in the stack thickness subspace^32^. **l** A PMUT as a receiver in the size-tension subspace^32^. **m** A PMUT as a receiver in the stack thickness subspace^32^. **n** A PMUT as a transceiver in the stack thickness subspace^32^. All pictures have been adapted with permission

PMUTs vibrate out-of-plane to transmit/receive sound, and they are generally fabricated in a circular geometry. At their fundamental frequency of vibration, the vibrating shape is best captured by a modified parabolic shape (Fig. 3a). Structurally, a PMUT comprises two important layers: the device layer and the piezoelectric-active layer (Fig. 3b). The device layer contains the neutral plane, with the centroid of each layer at a distance of *z*_*i*_ from the neutral plane. Each layer has a thickness, elasticity, prestress, density, and Poisson’s ratio of *h*_*i*_, *E*_*i*_, *σ*_*i*_, *ρ*_*i*_ and *ν*_*i*_, respectively. The radius of the device layer is *a*.

#### Basis for structural classification: the ‘Kappa-squared’

Structurally, PMUTs can behave as plates, membranes or both. A plate’s vibroacoustic response is determined by flexural rigidity (Fig. 3c), whereas a membrane’s response is determined by pretension (Fig. 3d). This behavior is determined by a nondimensional ratio of the product of the net structural pretension (*T*_*e*_) and square of *a* to the equivalent flexural rigidity (*D*_*e*_) of the PMUT ^32^.

$${\kappa }^{2}=\frac{{T}_{e}{a}^{2}}{{D}_{e}}$$

where

$${T}_{e}=\sum {\sigma }_{i}{h}_{i}$$

$${D}_{e}=\sum \left(\frac{{E}_{i}}{1-{{\nu }_{i}}^{2}}\right)\left(\frac{{{h}_{i}}^{3}}{12}+{{z}_{i}}^{2}{h}_{i}\right)$$

This expression indicates that a small, thick PMUT and a large, thin PMUT will demonstrate plate behavior and membrane behavior, respectively, at a constant *T*_*e*_^32^. Figure 3e presents the dependence of the nondimensional frequency parameter (${\alpha }_{00}{\beta }_{00}$) for the first mode of vibration (obtained while solving the PMUT’s natural response) on *κ*^2^, which is characterized by three zones. In the first zone, ${\alpha }_{00}{\beta }_{00}$ is constant with *κ*^2^ (0.1 to 1), demonstrating the plate behavior. Above 100, ${\alpha }_{00}{\beta }_{00}$ is linear with *κ*^2^ characterizing the membrane behavior. From 1 to 100, mixed behavior is observed. Figure 3f depicts the dependence of the maximum resonant deflection at the first mode of vibration (*w*_0_) on *a*, with a constant value of thickness and a constant *T*_*e*_. Three zones are observed with plate, plate–membrane, and membrane divisions. When describing PMUTs, it is important to learn about their dynamic responses, such as the natural response and the forced response. Many of these important expressions are tabulated in Fig. 3g.

#### Working principles of PMUTs: plate and membrane

The working principles of plate and membrane PMUTs^32^ are different. For a plate PMUT, applying direct current (DC) voltage across the piezoelectric layer tends to strain it due to the *d*_31_ piezoelectric effect. This strain is then restricted by the underlying device layer, leading to in-plane normal stress resultant *R* working from the piezoelectric layer’s centroid. Since plate PMUTs are thick, the neutral axis rests in the device layer (Fig. 3c, i). The difference (lever arm) between the piezoelectric layer’s centroid and the neutral axis, along with *R*, results in a bending moment *M* that bends the structure in an out-of-plane manner. Thus, applying an AC voltage makes the structure vibrate. Alternatively, for a membrane PMUT, the membrane is already stressed due to the presence of a net pretension in the structure, which, when coupled with structural inhomogeneity, causes the PMUT to bend without an electric field (Fig. 3d). A DC voltage induces a certain level of strain (*ε*) in the structure, which may occur due to the comparable thickness of the device and piezoelectric layer or due to a larger size-to-stack thickness ratio changing the level of tension (*τ*) in the structure, thereby changing the magnitude of bending. Applying an AC voltage makes the structure vibrate.

### PMUT functional classification

#### Lumped model of a PMUT

Functionally, a PMUT can be classified into three groups—transmitter, receiver, and transceiver—and a system-level model explains them the best. System-level models have been developed by several research groups^2,32,33^. The lumped parameter model couples the electrical domain and the mechano-acoustic domain with an ideal transformer, which represents piezoelectric electromechanical coupling. Figure 3h depicts the transmitter model, whereas Fig. 3i depicts the receiver model. The flow variable in the mechano-acoustic domain is the flow rate of the fluid volume displaced, represented as *q*. PMUT is a capacitor with capacitance *C*_*s*_. *V*_*in*_ and *P*_*in*_ are the input driving voltage and pressure, respectively, and *V*_*out*_ and *P*_*out*_ are the output receiving voltage and output pressure, respectively. *C*_*m*_, *R*_*m*_, *L*_*m*_ and *D*_*ac*_ are the structural compliance, damping, equivalent mass and acoustic impedance of the PMUT, respectively. *ϕ* is the turns ratio, representing the coupling between the electrical and mechano-acoustic domains.

#### PMUT as a transmitter

Design maps are available^32^ for the size–tension subspace and the thickness. In the size–tension subspace, a PMUT’s central deflection (*w*_0_) is proportional to the square of *a* in the plate regime, and it is almost independent of *a* in the membrane regime (Fig. 3j). Additionally, the iso-frequency lines (Fig. 3j) (black-dashed curves) suggest that low-residual tension devices have relatively high deflection sensitivities, even if they have to be shrunken to maintain the same operating frequency. Regarding thickness, the PMUT is ideally composed of passive (*h*_*passive*_) and device layers that add to a value of *h*_*sum*_. The maximum transmitted pressure (*P*_*TX*_) is plotted with respect to the thickness ratio *h*_*passive*_/*h*_*sum*_ (at *a* = 250 μm, *T*_*e*_ = 0 N/m). The optimal *h*_*passive*_/*h*_*sum*_ is reportedly 0.6 with pressure increasing according to the increase in overall PMUT thickness (Fig. 3k).

#### PMUT as a receiver

The PMUT as a receiver generates a charge (*Q*_*RX*_) that is directly proportional to *w*_0_^32^. In the size–tension subspace, both *w*_0_ and *Q*_*RX*_ increase as *a*^4^ (if *T*_*e*_ = 0) and as *a*^2^ if the PMUT is tension dominated; Fig. 3l). Additionally, the figure shows that the receiver at a lower operational frequency has higher deflection and charge output under a given layer configuration. In the thickness subspace, Q_*RX*_ is plotted with respect to *h*_*passive*_/*h*_*sum*_ (Fig. 3m). It is observed that *Q*_*RX*_ is directly proportional to *h*_*passive*_/*h*_*sum*_ and inversely proportional to the thickness of the piezoelectric layer for a given *h*_*sum*_. The plot also depicts that *Q*_*RX*_ decreases with *h*_*sum*_, which might be due to enhancement in the stiffness of the overall structure, thereby leading to reduced deflections and piezoelectric strain.

#### PMUT as a transceiver

The design map of a transceiver PMUT can be obtained by combining the effects of the transmitter and receiver^32^. Figure 3n represents the dependence of *Q*_*RX*_ on *h*_*passive*_/*h*_*sum*_ when the receiver is 1 m from the transmitter. The optimal thickness ratio to achieve the maximum *Q*_*RX*_ is reportedly 0.7.

### PMUT fabrication

#### Thin-film piezoelectric materials

**Fig. 4:**
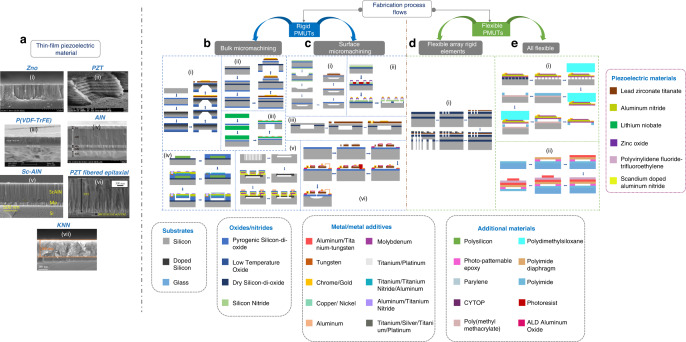
PMUT fabrication is divided into two parts: making the piezoelectric thin-film material and fabricating the PMUT structure with an internal piezoelectric thin-film by nanofabrication tools. **a** Various thin film piezoelectric material used to date: (i) zinc oxide (ZnO)^35^; (ii) lead zirconate titanate (PZT)^4^; (iii) polyvinylidene fluoride-trifluoro ethylene P(VDF)-TrFE^37^; (iv) aluminum nitride (AlN)^38^; (v) 15% scandium-doped AlN (Sc0.15AlN)^26^; (vi) PZT fibered epitaxial film^27^; and (vii) sodium potassium niobate (KNN)^42^. Fabrication methods divided into **b** PMUTs fabricated by bulk micromachining techniques: (i) wet etching of bulk silicon^43^; (ii) dry etching of bulk silicon^44^; and (iii) dry etching of bulk silicon with a thinned-down bulk ceramic^31,45^. **c** PMUTs fabricated by surface micromachining techniques: (i) prefabricated wet-etched trenches followed by wafer bonding^46^; (ii) surface release by wet etching^47^; (iii) cavity SOI and surface machining^48^; (iv) surface release by polysilicon wet etching^49^; (v) surface micromachining by silicon migration^50^; and (vi) surface release by wet etching^51^. **d** Flexible PMUT array with rigid elements: (i) fabrication of PMUT islands with connected springs^52^. **e** All flexible PMUTs: (i) flexible PMUTs made by PDMS stamp^53^ and (ii) flexible PMUT with flexible piezoelectric material on flexible substrate^29^. All pictures have been adapted with permission

PMUTs have been fabricated with several piezoelectric thin films, based on a popular contribution from Muralt et al.^34^ in which the transducers’ figure of merit from the thin-film materials perspective is mentioned. Some important materials are discussed in the following paragraphs, and their properties are tabulated in Table 1. The first thin-film piezoelectric material that was developed for PMUTs was the planar magnetron sputtered ZnO^35,36^ (Fig. 4a(i)), which is reported to exhibit excellent quality in terms of c-axis orientation with an X-ray rocking curve full width half maximum (FWHM) of less than 1°. The next popular ferroelectric material developed for PMUTs is the PZT (Fig. 4a(ii)). PZT has the highest piezoelectric constant (|e_31,*f*_| ~ 12 C/m^2^)^4^, thereby working best as a transmitter. However, PZT has a high dielectric constant (ε_33,*f*_ ~ 1200), making it unsuitable for manufacturing receivers. Next, P(VDF-TrFE)^37^ was developed (Fig. 4a(iii)) with the advantages of flexibility/stretchability and low-temperature process compatibility. The general deposition technique involves CSD of 10–15 wt.% copolymer pellets dissolved in methyl-ethyl-ketone, followed by low-temperature annealing. The fourth material is AlN (Fig. 4a(iv)), enabling PMUTs to be CMOS compatible, allowing monolithic integration of PMUTs with ASICs. Low-temperature sputtered AlN has a rocking curve FWHM of 3°, |e_31,*f*_| of 1.05 C/m^2^ and ε_33,*f*_ of 10.5 respectively^18,38,39^. AlN thin films are most suitable for PMUT receivers due to their small ε_33,*f*_. Next, Sc-AlN is developed with enhanced |e_31,*f*_|, thereby improving a PMUT’s overall performance. A 1 μm Sc_0.15_AlN film was developed by Wang et al.^26^ (Fig. 4a(v)), with a rocking curve FWHM of 1.9° for scandium doping of 15%. The |e_31,*f*_| was deduced indirectly from the frequency response of a Sc-AlN PMUT, and it was found to be 1.6 C/m^2^. Next, a PZT fiber epitaxial thin-film was developed by Thao et al.^27^ on oxide buffered layers by using magnetron sputtering followed by fast cooling (Fig. 4a(vi)). The thin-film exhibited a |e_31,*f*_| from 10 to 14 C/m^2^ and an ε_33,*f*_ from 200 to 300. Next, single crystal thin-film PZT^40^ and epitaxial thin-film PMnN-PZT^41^ were developed with |e_31,*f*_| values of 24 C/m^2^ and 14 C/m^2^ respectively. Recently, sodium potassium niobate (KNN) (Fig. 4a(vii)) was developed for making PMUTs^42^. KNN is lead-free and thus nontoxic, and it has better compatibility with CMOS. The layer was deposited following repetitive CSD followed by sessions of pyrolysis and annealing treatments to achieve an overall film thickness of 360 nm. The KNN thin-film demonstrated a |e_31,*f*_| of 8.5 to 14.4 C/m^2^ with an ε_33,*f*_ of 445.

**Table 1:** Figures of merit for various thin-film piezoelectric material used in PMUTs

Figures of merit	Notation	PZT^4^	PZT Fibered Epitaxial^27^	Single Crystal PZT^40^	Epitaxial PMnN-PZT^41^	AlN^18,38,39^	Sc_0.15_AlN^26^	ZnO^35,36^	KNN^42^
In-plane deflection force, piezoelectric charge in deformed PMUTs	|*e*_31,*f*_ | (C/m^2^)	8–12	14	24	14	1.05	1.6	1	8.5–14.4
Dielectric constant	*ε*_33,*f*_	300–1300	200–300	308	250	10.5	12	10.9	445
Piezoelectric voltage in deformed PMUTs	|*e*_31,*f*_ |/*ε*_0_*ε*_33_* (GV/m)	0.7–1.8	5.3–7.9	8.8	6.3	11.3	15	10.3	2.2–3.6
Coupling coefficient for flexural wave	*e*_31,*f*_^2^/*ε*_0_*ε*_33_* (GPa)	5.6–54.2	110–74	211	88	11.9	24	10.3	18.3–53
Out-of-plane piezoelectric force	*d*_33,*f*_ (pC/N)	60–130	–	–	–	3.9	–	5.9	–
Out-of-plane elastic constant	*c*_33_ (GPa)	98	–	–	–	395	–	208	–
Dielectric loss angle	tan *δ* (@ 1 to 10 kHz, 10^5^ V/m)	0.01–0.03	–	–	–	0.003	–	0.01–0.1	–
Signal-to-noise ratio	|*e*_31,*f*_ |/(*ε*_0_*ε*_33_tan *δ*)^0.5^ (Pa^0.5^)*	4–8	–	–	–	20	–	3–10	–

*Relative permittivity of free space (ε_0_): 8.85 pF/m

### Fabrication process flows

Fabrication-wise, a PMUT array can be broadly classified into rigid and flexible arrays. Rigid arrays have zero conformability and target applications that do not demand surface attachability, whereas flexible arrays can be partially to fully conformable.

#### Rigid PMUT arrays

##### Bulk micromachining

In this process, the bulk substrate is etched by deep etching methods to release the PMUT diaphragm. The first work^43^ in this direction began with a silicon wafer with pyrogenic oxide coated on the bottom surface, which was followed by boron diffusion, deposition of low-temperature oxide, backside oxide etches, and deep silicon wet etching by EDP to release the diaphragm. The top surface was coated with a metalized sol-gel PZT sandwich, which was wet etched to establish contacts (Fig. 4b(i)). The second work (Fig. 4b(ii)) began with a metalized and surface oxidized silicon-on-insulator (SOI) substrate, which was followed by top metal patterning, PZT/bottom metal, oxide, and device silicon etching to define the top structure, followed by deep reactive ion etching (DRIE) of the bulk silicon to release the membrane^44^. The third work^31,45^ began with a metalized bulk lithium niobate (LiNbO_3_) crystal that was wet oxidized. The assembly was then flipped and bonded to the surface of silicon and polished down to the thickness of the thin-film. The stack was deposited with metal on top, which was then patterned. DRIE was performed from the back to release the membrane (Fig. 4b(iii)).

##### Surface micromachining

In this process, the diaphragm is released by micromachining from the surface without etching the bulk silicon. The first work^46^ starts with a wet-oxidized silicon wafer that is coated with silicon nitride. A shallow trench is then defined in silicon by reactive ion etching (RIE). Another silicon wafer with an oxide coat facing downward is bonded to the previously machined wafer. The top silicon is thinned to a couple of microns, followed by PZT metal sandwich deposition, followed by top metal and PZT etching (Fig. 4c(i)). The second work (Fig. 4c(ii)) starts with a silicon wafer that is oxidized (low-temperature oxide), coated with nitride, and metalized. ZnO is then sputtered and etched along with the bottom metal and nitride to the desired pattern. Photoresist (PR) is pattern deposited in the trenches, and the resultant stack’s top surface is metalized. The stack is next patterned to create etch holes in the nitride layer. The oxide is then wet-etched to release the membrane^47^. The third work begins with a cavity SOI wafer (Fig. 4c(iii)), which is metalized and coated with PZT followed by its etch to take the ground connection, followed by the deposition and patterning of the top metal for live connection respectively^48^. The fourth work (Fig. 4c(iv)) starts with a silicon wafer deposited with a layer of patterned polysilicon. It is then covered with oxide and then coated with titanium/titanium nitride/aluminum after partial etching and filling with tungsten. AlN is next sputtered and then coated with a pattern of aluminum/titanium-nitride, followed by etching and sputtering to establish ground contact. The stack is patterned and etched, and the polysilicon sacrificial etched to release the membrane. The assembly is then coated with Parylene-C to seal off the cavity^49^. The fifth work starts with^50^ a silicon wafer with embedded shallow holes. The wafer is annealed to activate silicon migration, which forms a thin overlay over a cavity, forming a membrane. The membrane is next doped and deposited with patterned Sc-AlN and metal, followed by the pattern coat with oxide and metal. Finally, the released silicon membrane is pattern etched to define membranes of the desired shape and dimension (Fig. 4c(v)). The sixth work^51^ starts with an oxidized silicon wafer that is then coated with a patterned metal PZT sandwich. Aluminum oxide is then pattern deposited as shown in Fig. 4c(vi), which is followed by further metallization to define the in-device traces. PR is pattern-coated between the top metal and the bond pad, which is then pattern-sputtered with metal to establish the connection. The oxide attached to the silicon substrate is pattern etched, followed by a surface isotropic etch of the silicon to release the diaphragm.

#### Flexible PMUT arrays

##### Flexible array with rigid elements

In this category, the PMUT array is flexible, while each PMUT element is rigid. Sadeghpour et al.^52^ started with an oxidized SOI substrate, which is metalized and pattern etched in a desired fashion. PZT is next pattern deposited, followed by further patterned oxidization and metallization to establish top contacts. A subsequent DRIE is performed to etch the bulk silicon to release the membrane, followed by a patterned RIE to desire etch silicon from the top to create free serpentines (Fig. 4d(i)).

##### All flexible PMUTs

In this category, both the PMUT elements and arrays are flexible. The first work starts with a layered stack, as shown in Fig. 4e(i). A PDMS stamp is then used to remove the functional layers and transfer them to another stack with silicon, poly(methylmethacrylate), and patterned polyimide. Poly(methylmethacrylate) is then stripped off to detach and create the flexible PMUT^53^. The second work begins with a polyimide-on-glass substrate, which is patterned with a photopatternable epoxy (Fig. 4e(ii)). A polyimide diaphragm is suspended on top of it, which is followed by the deposition of a metal PVDF sandwich. The stack is then detached from the glass substrate to create flexibility ^29^.

### Author’s sectional predictions

In the last decade, at least 5 new materials have been developed for PMUTs, with single crystal PZT and Sc-AlN being the most effective options for transmitters and receivers, respectively. Future trends show that 5 more new materials are to be developed in the next 5 years with improved transmit–receive efficiencies; scholars will focus on developing transparent and flexible thin films. While fabrication has seen many developments, rigid PMUTs have reached a saturation point. However, flexible/stretchable PMUT arrays still have room for improvement, with more than 5 new flows predicted in this direction within 2 years.

## Special PMUTs

In this section, structurally and functionally nonconventional PMUTs are described, which are broadly classified into structurally modified PMUTs and flexible PMUTs.

## Structurally modified PMUTs

**Fig. 5:**
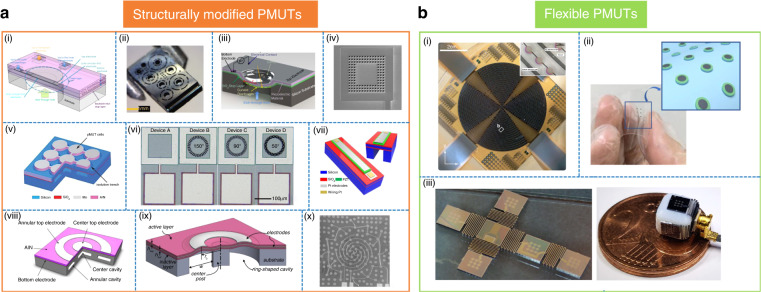
Special PMUTs which can be classified as follows. **a** Structurally modified PMUTs: (i) bimorph PMUT with enhanced transmit sensitivity^54^; (ii) PMUT with venting rings and enhanced transmit sensitivity^55^; (iii) curved PMUT with increased pressure output^56^; (iv) PMUT with relaxed boundary conditions^57^; (v) PMUT with isolation trench and enhanced sensitivity^60^; (vi) PMUTs with V-shaped rings^61^; (vii) broadband PMUTs^62^; (viii) central and annular PMUTs on a single chip^65^; (ix) ring-shaped PMUT^66^; and (x) Archimedean PMUT array^67^. **b** Flexible PMUTs: (i) polymer PMUT^72^; (ii) fully flexible PMUT^53^; and (iii) flexible PMUT die with rigid elements^52^. All pictures have been adapted with permission

PMUTs have been structurally modified to enhance their performance levels in terms of various parameters, including deflection/transmit sensitivity, directivity, and bandwidth. Akhbari et al. fabricated bimorph AlN PMUTs with two piezoelectric layers in an array, as shown in Fig. 5a(i). The scholars claim that their PMUTs have four times the electromechanical coupling coefficient relative to unimorph AlN PMUTs of similar geometry and frequency^54^. Rozen et al. fabricated PMUTs with venting rings (Fig. 5a(ii)) to amplify the far-field sound pressure level (SPL). An increase of 4.5 dB over a control device was claimed^55^. Akhbari et al. developed a curved PMUT (Fig. 5a(iii)) with a radius of curvature of 400–2000 μm and a deflection sensitivity that was 50 times greater than that of a comparable flat device^56^. Wang et al. etched holes along the periphery of a PMUT (Fig. 5a(iv)), which increased the SPL by 5.3 dB compared with a similar device^57–59^. Wang et al. created isolation trenches along the PMUT periphery (Fig. 5a(v)), which increased the output pressure by 76% compared with a similar device. The authors claimed that the trench reduced the deflection-induced tensile membrane stress, allowing for more motion^60^. Chen et al. reported V-shaped surface springs (Fig. 5a(vi)), which increased the deflection sensitivity by 203% due to the release of residual stress from the relatively flat membrane^61^. Wang et al. fabricated a wide-frequency band rectangular PMUT by using the technique of mode merging (Fig. 5a(vii)). The -6 dB bandwidth was reported to be 95% in water, which was exceptionally higher than that of the control device^62–64^. Wang et al. fabricated a combination of central and annular PMUT-on-a-single-chip (Fig. 5a(viii)), which increased the transmit and receive sensitivities by 1.9 and 6.5 times those of the control PMUT, respectively. The scholars claimed that the phenomenon was caused by the coupling between the two separate membranes^65^. Eovino et al. reported on a ring-shaped PMUT (Fig. 5a(ix)) with a central post, and they observed that the velocity bandwidth in fluid-coupled operations reached 160%, which was claimed to be 60% greater than any other reported bandwidth. The researchers claimed that the ring geometry and acoustic-induced resonance caused the broadband nature^66^. Wang et al. reported on a spiral Archimedean PMUT array (Fig. 5a(x)), which they claimed could generate 18% higher sound pressure than conventional phased PMUT arrays with similar dimensions ^67^.

### Flexible PMUTs

There has been a recent surge in wearable transducer development^68–71^. PMUTs have been make-wise modified to develop flexible arrays with the added advantages of wearability and conformability to various surface topologies. Chare et al. fabricated large-area PMUTs based on flexible PVDF (Fig. 5b(i)) for in-air haptics and demonstrated their applicability in display compatible flat arrays^72^. Although the array was fabricated on a glass substrate, the same group reported fully flexible arrays^29^. Sun et al. reported a PMUT array sticker (Fig. 5b(ii)), which they found by using PDMS stamp-based transfer. The devices had resonant frequencies and displacement sensitivities of 2.58 MHz and 30 nm/V, respectively^53^. Sadeghpour et al. created bendable PMUT arrays (Fig. 5b(iii)) that were connected by silicon springs; they demonstrated the array’s capabilities by wrapping it around a 3D cube with dimensions of 5 mm and a bendability of 90° ^52^.

## PMUT applications

PMUT applications can be classified as transmitters, receivers, and transceivers, as described below; some important comparisons of these terms are tabulated in Table 2.

**Table 2:** Application classified PMUT performance comparison

Application category	Piezoelectric material	PMUT geometry	PMUT lateral dimension^a^	PMUT stack thickness (st + ac) (μm)^b^	Array configuration	Operating frequency	Deflection sensitivity	Transmit Sensitivity	Receive/ Sensing Sensitivity	BW^c^ @ -6 dB	Applied for
Transmitter	PZT	Rectangular	(l × w): 8.3 mm × 680 μm	5 + 1.5	32 channels, linear array	1.4 MHz in water	–	45.9 kPa/V, @ 20 mm (focused)	–	–	Phased array for neuromodulation^73^
	AlN	Annular, concentric	w: 60 μm	5 + 0.83	5 channels	6 MHz in mineral oil	–	12 kPa/V @ 1.9 mm (focused)	–	–	Proposed phased array for catheter HIFU^74^
	PZT	Circular	a: 120 μm	3.7 + 1	10 × 29 channels	1.5 MHz in culture medium	–	30 kPa/V @ 1 mm (unfocused)	–	–	LIPUS for cell stimulation^75^
	PVDF	Circular	a: 250 μm	10 + 0.45	22 annular channels	229 kHz in air	–	21 Pa/V @ 30 mm (focused)	–	–	Haptic feedback^72^
	PZT	Circular	a: 60 μm	4 + 1.9	–	8 MHz in water	40 nm/V @ 6.8 MHz	1.9 kPa/V @ 7.5 mm (unfocused)	–	62.5%	Particle manipulation^77^
Receiver	PZT	Circular	a: 60 μm	4 + 1.9	65 channels, Linear array	6.75 MHz in water	–	3.24 kPa/V @ 7.5 mm (unfocused)	0.48 mV/kPa @ 0 dB gain	89%	Photoacoustic imaging^81^
	AlN	Circular	a: 92 μm	1.45 + 1	10 × 10 channels	700 kHz in air	–	–	–	–	Zero power ultrasound receiver^90^
	PZT	Rectangular	(l × w): 500 μm × 250 μm	11 + 2	Single channel	285 kHz in water	–	–	2 mV/mW/cm^2^	74%	Energy harvesting^25^
Transceiver	PZT	Rectangular	(l × w): 110 μm × 80 μm	6 + 1	512 channels, (32 × 16)	5 MHz in tissue	–	–	–	30%	Intravascular ultrasound^92^
	AlN	Rectangular	(l × w): 58 μm × 43 μm	1.2 + 1	6160 channels, (110 × 56)	14 MHz in air	–	625 Pa/V @ 250 μm (focused)	344 μV/kPa	33% in PDMS	3D fingerprint sensor^93^
	PZT	Circular	a: 750 μm	10 + 0.5	1 channel	235 kHz in air	1.5 μm/V	–	0.56 mV/μm	–	Fluid density sensor^30^
	Single-crystal PZT	Circular	a: 1250 μm	6.25 + 2	4 channels	40–50 kHz in air	10.75 μm/V	0.4 Pa/V @ 33 cm (unfocused)	2.8 mV/Pa	11%	Long rangefinder^40^
	LiNbO_3_	Rectangular	(l × w): 170 μm × 130 μm	1.35 + 1	1 channel, (15 × 15)	630 kHz in water	80 nm/V in air	–	–	64%	Intrabody communication^31^

^a^*l*: length, *w*: width, *a*: radius

^b^*st*: structural layer thickness, *ac*: active layer thickness

^c^*BW*: Bandwidth

### PMUTs as transmitters

**Fig. 6:**
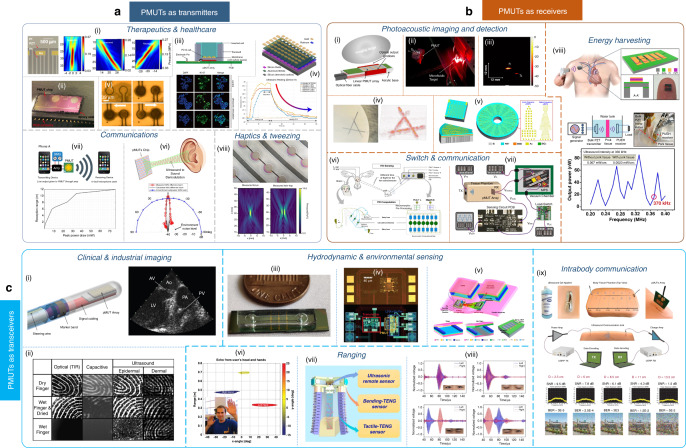
PMUT applications. **a** PMUTs as transmitters: (i) 32 element linear phased array for neuromodulation^73^; (ii) annular phased array for HIFU^74^; (iii) PMUTs for cell stimulation^75^; (iv) PMUT phased array for bioheating^76^; (v) PMUTs applied to acoustofluidics for particle streaming^77^; (vi) PMUTs for directional ultrasound^78^; (vii) near-ultrasound PMUTs for in-air communication^79^; and (viii) PVDF PMUT arrays for in-air focusing^72^. **b** PMUTs as receivers: (i) PA system using PMUTs^81^ (ii) photoacoustic spectroscopy system for concentration detection^83^; (iii) single-cell PMUT for low-frequency PA imaging^84^; (iv) PMUTs operating at high modes for PA imaging application^85^; (v) thick-film PMUTs for multifrequency imaging^86^; (vi) neuromorphic object localization with PMUTs^89^; (vii) PMUTs as zero-powered wake-up switches for communication^90^; and (viii) broadband energy harvester using PMUT^25^. **c** PMUTs as transceivers: (i) IVUS intracardiac imaging system using high-density PMUT arrays^92^; (ii) 3D ultrasound on chip by using PMUTs with ASICs^93^; (iii) PMUT microfluidic integration for fluid density sensing^30^; (iv) single-cell PMUT on a chip for hydrodynamic property sensing^100^; (v) TENG powered PMUTs for gas-sensing applications^28^; (vi) in-air gesture recognition system by using AlN PMUTs^101^; (vii) AI-enabled PMUT integrated soft robotics^102^; (viii) eye blinking system by using PMUTs^103^; and (ix) PMUTs for intrabody communication^31^. All pictures have been adapted with permission

As transmitters, PMUTs generate sound and have been applied to the domains of therapeutics, health care, communications, and haptics. Regarding therapeutics, Tipsawat et al.^73^ developed a 32-element phased array PMUT with beam steering by using Nb-doped PZT for neuromodulation (Fig. 6a(i)). An acoustic pressure of 0.44 MPa was obtained at a focal distance of 20 mm with an average intensity of 1.29 W/cm^2^. Eovino et al. reported on concentric PMUT arrays^74^ for focused ultrasound applications that could focus on a spot of 1.9 mm for a pressure of 12 kPa/V (Fig. 6a(ii)). Lee et al. created a low-intensity pulsed ultrasound system^75^ by using a PMUT linear array that generated a pressure of 0.15 MPa at 1 mm; they increased the cell proliferation rate in the range of 138–166% with respect to the control condition (Fig. 6a(iii)). Pop et al. created a bioheating platform^76^ with a 5 ×10 PMUT phased array and demonstrated a 4 °C increase in the relative temperature after 10 s (Fig. 6a(iv)). In acoustofluidics, Cheng et al.^77^ developed confined PMUT arrays to trap and manipulate 4-μm silica beads using unipolar excitation (Fig. 6a(v)). In communications, Shao et al.^78^ reported on a parametric air-coupled single-chip bimorph PMUT array to generate highly directional audible sound with a half-power beam width that was less than 5°. The scholars generated a 5-kHz sound by combining frequencies of 252 and 257 kHz (Fig. 6a(vi)). Harshvardhan et al.^79,80^ developed near-ultrasound PMUTs for sending data over sound. PMUTs could send data successfully for a range of 6 m while consuming less than 10 mW of electrical power (Fig. 5a(vii)). In haptics, Chare et al.^72^ demonstrated a large-area thin-film PVDF-based PMUT array (Fig. 6a(viii)), which could display the creation of a twin trap that reached an acoustic pressure of 1.6 kPa after 20 mm from the PMUT in air.

### PMUTs as receivers

As receivers, PMUTs receive sound and are applicable to photoacoustic (PA) imaging, PA detection, switching, communication, and energy harvesting. In PA imaging, Dangi et al.^81,82^ combined a PMUT array with a pulsed light source in a single device that could image a custom-made phantom with embedded lead targets, as shown in Fig. 6b(i). The PMUTs in the device had center frequencies of 6.75 MHz in water with a PA bandwidth of 89%. Next, Roy et al.^83^ developed a PMUT-based pulsed PA microfluidic concentration detector that was capable of detecting ink-water concentrations, followed by the development of a single-cell low-frequency PMUT that could image targets clearly in a tissue-mimicking phantom^84^ (Fig. 6b(ii),(iii)). Recently, Cai et al.^85^ made a high-order multiband AlN PMUT and used its high modes to obtain enhanced PA images from a custom-made phantom (Fig. 6b(iv)). The scholars used BRISQUE as an image quantifier and observed that the higher modes of PMUTs provided better images than the lower modes at similar depths. Zheng et al.^86^ developed a thin ceramic PZT for multifrequency PA imaging. Although this device involved a relatively thick-film (9 μm) PZT, the work was considered novel and was thus reported in this review (Fig. 6b(v)). Recently, Wang et al. demonstrated the usage of AlN and Sc-AlN PMUTs for microwave-induced thermoacoustic imaging applications^87,88^. In switch and communication applications, Moro et al.^89^ developed a neuromorphic object localization system that used PMUTs and resistive memories to accomplish the work (Fig. 6b(vi)). Pop et al.^90^ developed a zero-power PMUT-based ultrasonic wake-up receiver, a zero-power MEMS plasmonic switch, and a low-leakage current CMOS load switch as important parts of the system. A working demonstration of the wake-up behavior was shown in a range of 5 cm (Fig. 6b(vii)). In the domain of energy harvesting^91^, Shi et al.^25^ demonstrated making a rectangular PMUT-based broadband energy harvester for self-powered implantable medical devices (IMDs) (Fig. 6b(viii)). The scholars demonstrated power transfer through a 6 mm pork tissue in water to receive a power of 2.3 µW/cm^2^.

### PMUTs as transceivers

As transceivers, PMUTs transmit and receive sound; additionally, they can function with a differential actuation-sensing scheme as frequency-shift devices or can function as a transmit–receive pair with device twins facing each other. In the first direction, Dausch et al.^92^ fabricated two 256- and 512-element arrays through silicon vias to make an intracardiac catheter that captured a B-mode image in vivo from a porcine model (Fig. 6c(i)). Next, Tang et al.^93^ proposed a 3-D ultrasonic fingerprint sensor on-chip consisting of a 110 ×56 PMUT array bonded to a custom 180 nm readout ASIC and can image epidermal, subsurface, and dermal fingerprints (Fig. 6c(ii)). Next, for frequency shift applications^94–97^, Roy et al. devised a PMUT-microfluidic integrated device^98,99^ to sense fluid density; they demonstrated real-time density monitoring with the device^30^ (Fig. 6c(iii)). Ledesma et al.^100^ developed a single-cell PMUT on a 130 nm CMOS chip to monitor fluid properties, such as density, viscosity, and sound compressibility (Fig. 6c(iv)). Next, Sun et al.^28^ constructed a TENG-powered functionalized PMUT to demonstrate its functionality as a combined temperature and humidity sensor (Fig. 6c(v)). Next, Przybyla et al.^101^ made a 3D on-chip rangefinder to localize targets over a 90° field of view to a 1-m distance (Fig. 6c(vi)). Shi et al.^102^ made an AI-enabled soft robotic perception system with PMUT-based auto positioning and TENG to achieve appropriate positioning of randomly distributed objects (Fig. 6c(vii)). Sun et al.^103^ worked on a portable eye-blinking monitoring application by mounting a similar PMUT on a spectacle that could track eye blinks (Fig. 6c(viii)). As transmit–receive pairs, PMUTs are applicable in intrabody communications; Pop et al.^31^ created a special in-plane actuated PMUT using an anisotropic LiNbO_3_ thin-film for enhanced bandwidth characteristics. Image data were sent 13.5 cm through the body tissue phantom (Fig. 6c(ix)).

### Author’s sectional predictions: PMUTs and their competitors

PMUTs have wide-ranging applications in health care, consumer electronics, and industrial automation. As transmitters, they are used in low-intensity focused ultrasound for neuromodulation, showing potential for achieving greater acoustic intensity efficiency than CMUTs and bulk piezoelectric transducers. As receivers, PMUTs are applied in PA, offering advantages in making high fill-factor multifunctional arrays in any shape that are integrate-able into CMOS. Although a PMUT–PA tomography system has yet to be developed, initial results from synthetic aperture PA imaging are promising. As transceivers, PMUTs are successful as low-voltage rangefinders and fingerprint sensors. In medical imaging applications, PMUTs suffer from reduced axial resolution due to their high Q factors; however, improving backing layer schemes in PMUTs can supposedly significantly enhance PMUT imaging performance.

## Conclusion

This concise review thus forms an all-in-one reference for the important works conducted since the beginning of PMUT manufacturing, and it provides readers with an overall awareness of PMUTs, their history, their present progress, their design, and their potentials for various applications. Although PMUTs have brought about the new ultrasound revolution, similar to any other technologies, they have their advantages and disadvantages. The advantages have been discussed earlier. Some of the disadvantages over traditional piezoelectric transducers are the limitation preventing the creation of high-intensity ultrasound, the relatively complicated backend, and the high investment cost in terms of capital and time for new ventures. Despite these limitations, it is believed that similar to all technologies, PMUTs should continue to survive and prosper for years to come, and they should be applied by fellow researchers and entrepreneurs to develop meaningful applications suitable for driving the MEMS ultrasound revolution.
